# A Single-Center Retrospective Study on Noninvasive Prediction of Terson Syndrome in Aneurysmal Subarachnoid Hemorrhage (aSAH) Patients: The Role of CT-Measured Posterior Globe Thickness and Age

**DOI:** 10.7759/cureus.97061

**Published:** 2025-11-17

**Authors:** Yuki Hayashi, Takumi Kitamura, Ryusei Seo, Shusaku Matsuo, Takeshi Torigai

**Affiliations:** 1 Neurosurgery, Chutoen General Medical Center, Kakegawa, JPN

**Keywords:** aneurysmal subarachnoid hemorrhage (asah), noninvasive imaging modalities, posterior globe thickness, retinal hemorrhage, single-center retrospective study, terson syndrome, vitreous hemorrhage

## Abstract

Background

Terson syndrome (TS), an intraocular hemorrhage secondary to aneurysmal subarachnoid hemorrhage (aSAH), has a high incidence rate. Clinically, patients with aSAH often present with concomitant TS; however, owing to the difficulty in performing ophthalmic examinations in critically ill patients, many cases may be missed. This study aimed to develop and evaluate a CT-based diagnostic model incorporating posterior globe thickness to predict TS in patients with aSAH.

Materials and methods

This was a retrospective study on patients who underwent direct surgery or endovascular treatment for ruptured cerebral aneurysms at our institution between January 1, 2018, and August 31, 2025 (analyzed by eye). We extracted data from eyes definitively diagnosed with TS via ophthalmic examination. In addition to collecting epidemiological and clinical data, posterior globe thickness was measured for each eye. Statistical analyses included the Mann-Whitney U test, chi-square test, generalized estimating equation (GEE) logistic regression analysis, and receiver operating characteristic (ROC) analysis. Statistical significance was set at p < 0.05.

Results

A total of 177 patients (354 eyes) received aSAH treatment, of whom 26 individuals (52 eyes) underwent ophthalmic examination, and within this subgroup, 11 patients (17 eyes) were diagnosed with TS. In the univariate GEE logistic regression analysis, the presence of TS was significantly correlated with age (p=0.005), World Federation of Neurosurgical Societies (WFNS) grade (p=0.021), complaints of visual and visual field impairment (p=0.021), and posterior globe thickness (p=0.038). The multivariate GEE logistic regression analysis demonstrated that age and posterior globe thickness significantly influenced the risk of developing TS. In this final multivariate model, the odds of having TS decreased by a factor of 0.85 for every one-year increase in age (p=0.007), whereas the odds increased by a factor of 13.74 for every 1 mm increase in posterior globe thickness (p=0.027). ROC analysis, performed using this final multivariate model, yielded a calculation to determine the age-dependent cutoff for posterior globe thickness: Cutoff(mm)≈−1.295+0.0637×Age (years), which showed a sensitivity and specificity of 82.4% and 82.9%, respectively.

Conclusion

This study proposes a noninvasive prediction model for estimating TS based on CT measurements of posterior globe thickness. Serving as a practical triage tool, these findings suggest that incorporating age significantly enhances the diagnostic utility. To ensure broad generalizability and facilitate its application in clinical practice, prospective multicenter trials are necessary to validate these results.

## Introduction

Aneurysmal subarachnoid hemorrhage (aSAH) can lead to a spectrum of complications, including Terson syndrome (TS). The association between SAH and retinal hemorrhage was first described by Litten in 1881. In 1900, Albert Terson reported a link between SAH and vitreous hemorrhage, leading to the eponymous "Terson's syndrome" [1,2]. Subsequently, as it became clear that intraocular hemorrhages could also occur in the subretinal and subvitreous space [3], the definition of TS was expanded to include these areas. Currently, neurosurgeons recognize TS as an intraocular hemorrhage associated with an acute increase in the intracranial pressure caused by aSAH. The reported prevalence of TS in patients with aSAH ranges from 6% to 32% [4-11]. Furthermore, TS is also associated with other neurological pathologies, including traumatic brain injury and intracerebral hemorrhage, underscoring its relevance to neurosurgical practice [3,12,13]. Despite this clear association, this condition remains underdiagnosed or overlooked in routine clinical practice.

TS is typically diagnosed using specialized ophthalmological examinations, including fundoscopy, ocular ultrasonography, and optical coherence tomography [7,14,15]. However, subjecting every patient with aSAH to ophthalmological assessment is often not feasible in real-world clinical settings. Some patients die during the course of the disease because of its severity, whereas others are unable to report symptoms because of impaired consciousness or other reasons. Because the orbits are routinely included in initial head computed tomography (CT) scans for diagnosing aSAH, some studies have suggested that crescent-shaped or nodular hyperdensities in the posterior globe on CT images can serve as diagnostic aids for TS [16,17]. However, other reports have indicated that the diagnostic accuracy of CT is inferior to that of formal ophthalmologic examinations [14]. If neurosurgeons could readily identify patients at high risk of TS, timely ophthalmologic consultation would be facilitated. Early intervention could prevent the deterioration of visual function, enhance the effectiveness of rehabilitation, and ultimately improve patient prognosis [13].

The primary objective of this study was to derive and internally validate a non-invasive, CT-based prediction model for TS in patients with aSAH. This model specifically utilizes CT-measured posterior globe thickness and patient age as key predictors. Ultimately, the study aimed to use the validated model to propose an age-adjusted diagnostic threshold for posterior globe thickness, serving as a triage tool to accurately guide and prioritize patients requiring formal ophthalmologic evaluation for TS.

## Materials and methods

Study design and population

This retrospective cohort study was conducted at our institution and included 177 patients (354 eyes) who were admitted for aSAH between January 1, 2018, and August 31, 2025. The term "aSAH" in this study included both saccular and dissecting aneurysms. All included patients underwent either direct or endovascular treatment for the aneurysm.

From this initial cohort, we extracted data from a subgroup of 26 patients (52 eyes) who underwent a comprehensive ophthalmological evaluation. This evaluation was initiated either because of patient-reported visual symptoms or at the discretion of the attending medical staff who deemed it necessary. The presence or absence of TS, which was defined as any form of intraocular hemorrhage (including preretinal, retinal, subretinal, vitreous, or subvitreous hemorrhages), was confirmed by a staff ophthalmologist using fundoscopic examinations. This examiner was blinded to the posterior globe thickness measurements derived from CT.

Data collection

Patient-level data, including age, World Federation of Neurological Surgeons (WFNS) grade, presence of seizures, and aneurysm location (anterior or posterior circulation), were retrieved from electronic medical records. For ophthalmological analysis, each eye was treated as a separate unit, resulting in 52 eyes for analysis. Statistical models accounted for the intraclass correlation between the two eyes of the same patient.

**Figure 1:**
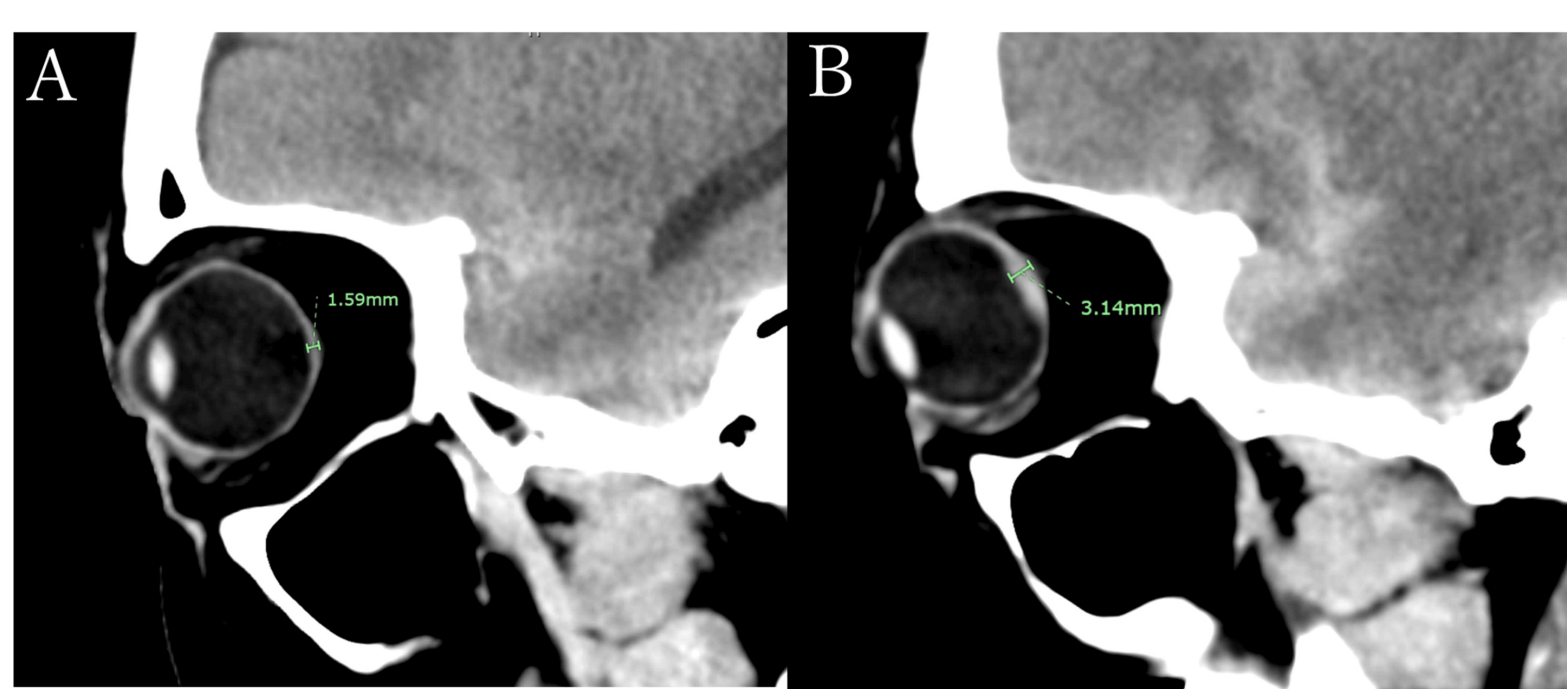
Measurement of posterior globe thickness on sagittal head CT. The thickness of the posterior globe was measured perpendicularly using a digital ruler on the sagittal view of the initial head computed tomography (CT) scan. The reference slice was defined as the one where the crystalline lens was maximally visualized, and the measurement was taken at the thickest hyperdense portion of the posterior wall of the globe. The unit of measure is in mm (millimeters). A: Retinal hemorrhage in a 29-year-old woman with subarachnoid hemorrhage due to a ruptured left internal carotid artery aneurysm. B: Vitreous hemorrhage in a 41-year-old woman with subarachnoid hemorrhage due to a ruptured anterior communicating artery aneurysm.

The thickness of the posterior globe was measured using initial (pretreatment) head CT. The CT scans were performed using a Siemens SOMATOM Definition Flash scanner (Siemens Healthcare, Forchheim, Germany or a SOMATOM Definition AS+ when concurrent CT angiography was performed). The parameters included a tube voltage of 120 kVp, a tube current adjusted according to patient physique (ranging approximately from 200 to 300 mA), a slice thickness of 5 mm, a window level of 35 Hounsfield Units (HU), and a window width of 80 HU. The sagittal view was used to identify the slice in which the crystalline lens was maximally visualized. For this reference slice, the thickest hyperdense portion of the posterior wall of the globe was measured perpendicularly using a digital ruler. The measurement procedure is illustrated in Figure 1. This measurement protocol was applied consistently in all the cases. To ensure measurement reliability, all assessments were performed by the first author, who was blinded to the TS diagnosis. Interrater reliability was confirmed by recalculating the measurements for 10 randomly selected CT scans, and the intraclass correlation coefficient (ICC) was computed. The presence of subjective visual complaints, specifically visual acuity or visual field disturbances, was determined from chart reviews prior to the ophthalmology consultation and recorded on a per-eye basis. For instance, if a patient reported visual loss only in the right eye, the left eye was recorded as having no complaints. Eyes were categorized as having "no complaint" if the symptoms were other than visual acuity/field loss (e.g., diplopia, eye discharge) or if ophthalmologic consultation was initiated based on the physician's judgment alone.

Statistical analysis

All statistical analyses were performed using IBM SPSS Statistics for Windows, Version 29 (Released 2023; IBM Corp., Armonk, New York, United States).

Baseline comparison**

To assess potential selection bias, we compared the baseline characteristics of the analyzed group (n=26, who underwent eye examinations) and the non-analyzed group (n=151, who did not). We used established prognostic factors for SAH severity, including age, WFNS grade (categorized as low grade I-III vs. high grade IV-V), modified Fisher grade (low risk, 0-2 vs. high risk, 3-4), presence of seizures, and aneurysm location (anterior vs. posterior). The Mann-Whitney U test was used for continuous variables, and the chi-square test was used for categorical variables. In addition, we compared the baseline characteristics of the TS-positive and TS-negative eye groups.

Optimization of the TS prediction model via univariate and multivariate analyses

Univariate Analysis

Univariate generalized estimating equation (GEE) logistic regression analyses were performed to examine the association between TS and several predictors. These included age, WFNS grade, modified Fisher grade, seizures, aneurysm location, subjective visual complaints, and computed tomography-measured posterior globe thickness. The GEE model was specified using an exchangeable working correlation matrix to adjust for the correlation between the two eyes of each patient. Robust standard errors were used for all GEE analyses to provide valid inference regardless of the specified correlation structure. Continuous variables (age and posterior globe thickness) were entered into the model as linear variables. The WFNS and modified Fisher grades were dichotomized as described above.

Multivariate Analysis and Model Selection

Multivariate GEE logistic regression analysis was conducted to identify independent predictors of TS. The model again utilized an "exchangeable" correlation matrix with the patient ID as the subject variable. Adhering to the events per variable (EPV) criterion, we included all variables from the univariate analysis that showed a p-value of less than 0.20 in the initial multivariate model.

Prior to this analysis, multicollinearity among the predictor variables was assessed by calculating the variance inflation factor (VIF) in a standard linear regression model. Variable combinations with a VIF exceeding 5 were excluded. The final predictive model was derived using a backward elimination approach based on the Wald χ² test, retaining only the statistically significant independent predictors (p < 0.05). Quasi-likelihood under the Independence model criterion (QIC) was used as a supplementary measure to aid model fit assessment during the selection process.

Sensitivity analysis

To assess the robustness of the eye-level modeling and address the potential issue of clustering, a patient-level logistic regression analysis was conducted as a sensitivity check. The analysis was restricted to the n=26 patients who underwent eye examinations, using the endpoint "any-eye TS present (yes/no)." Predictors identified in the final multivariate GEE model were included, with posterior globe thickness represented by the mean measurement of both eyes.

Evaluation of diagnostic performance and cutoff determination

ROC curve analysis was performed to evaluate the diagnostic utility of two models for predicting TS: the Univariable Model using only posterior globe thickness as the predictor and the Final Multivariate Model-with the optimal predicted probability for both determined by the point that maximized Youden's Index (Sensitivity + Specificity − 1); the corresponding posterior globe thickness measurement for each optimal predicted probability was estimated using the model's regression equation, whereby for the univariable model, the thickness cutoff was directly back-calculated by substituting the predicted probability to determine the final sensitivity and specificity for this estimated thickness cutoff value, whereas for the final multivariate model, the sensitivity and specificity corresponding to the optimal predicted probability were calculated, and the mathematical formula was presented to allow the corresponding thickness value to be algebraically derived by substituting the predicted probability and the only remaining continuous variable, age, into the regression equation.

Statistical significance was set at p < 0.05 for all tests.

Ethical considerations

This study was conducted in compliance with the ethical guidelines of our institution and was approved by the Institutional Review Board (Approval No. 1326251002). The requirement for written informed consent was waived because all patient data were fully anonymized during collection and analysis.

## Results

Patient/ocular characteristics and population prevalence of TS

The study population comprised 177 patients with aSAH (354 eyes) who were stratified into the analyzed group (n=26, patients who underwent eye examination) and the non-analyzed group (n=151, patients who did not undergo eye examination). Among the 26 patients analyzed (52 eyes), 11 (6.2% of the total cohort) were diagnosed with TS, resulting in 17 TS-positive eyes (4.8% of the total cohort). Comparisons of baseline characteristics between the analyzed and non-analyzed patient groups and between the TS-positive and TS-negative eye groups are shown in Tables 1, 2, respectively. Intra-rater reliability for the measurement of posterior globe thickness was assessed by a single experienced observer measuring 10 eyes on two separate occasions separated by an interval of more than 10 days. The interrater reliability was determined to be ICC>0.75.

**Table 1:** Baseline characteristics of the analyzed and non-analyzed patient groups. Data for age are presented as Mean±SD (Standard Deviation), and all other variables are presented as N (number of patients) (%). Statistical significance was set at p < 0.05 for all tests. WFNS: World Federation of Neurosurgical Societies

Characteristic	Analyzed Group (n=26)	Non-analyzed Group (n=151)	p-value
Age (mean ± SD), years	53.1 (±12.3)	62.7 (±12.4)	<0.001
WFNS Grade			0.262
Low Grade (I–III), n (%)	15 (57.7)	104 (68.9)	
High Grade (IV–V), n (%)	11 (42.3)	47 (31.1)	
Modified Fisher Scale			0.940
Low Risk (0–2), n (%)	5 (19.2)	30 (19.9)	
High Risk (3–4), n (%)	21 (80.8)	121 (80.1)	
Seizures (n (%))	2 (7.7)	11 (7.3)	0.941
Aneurysm Location			0.306
Anterior Circulation, n (%)	21 (80.8)	133 (88.1)	
Posterior Circulation, n (%)	5 (19.2)	18 (11.9)	

**Table 2:** Baseline characteristics of the TS-positive eyes and TS-negative eye groups. Data for age and posterior globe thickness are presented as mean ± SD (standard deviation), and all other variables (WFNS Grade, Modified Fisher Scale, presence of seizures, aneurysm location, and complaint of visual/visual field impairment) are presented as N (number of eyes) (%). Statistical significance was set at p < 0.05 for all tests. †p<0.05 (based on univariate GEE logistic regression analysis). TS: Terson syndrome; WFNS: World Federation of Neurosurgical Societies

Characteristic	TS-Positive Eyes (n=17)	TS-Negative Eyes (n=35)	Statistical Comparison (p-value)
Age (mean ± SD)	45.6±9.2	56.7±11.9	0.005†
WFNS Grade			0.021†
Low Grade (I–III), n (%)	5 (29.4)	25 (71.4)	
High Grade (IV–V), n (%)	12 (70.6)	10 (28.6)	
Modified Fisher Scale			0.500
Low Risk (0–2), n (%)	2 (11.8)	8 (22.9)	
High Risk (3–4), n (%)	15 (88.2)	27 (77.1)	
Presence of Seizures (n (%))	3 (17.6)	1 (2.9)	0.233
Aneurysm Location			0.870
Anterior Circulation, n (%)	14 (82.4)	30 (85.7)	
Posterior Circulation, n (%)	3 (17.6)	5 (14.3)	
Complaint of Visual/Visual Field Impairment (n (%))	7 (41.2)	8 (22.9)	0.021†
Posterior Globe Thickness (mean ± SD, mm)	2.27±0.71	1.59±0.28	0.038†

Univariate GEE logistic regression analysis

The results of the univariate GEE logistic regression analysis, which assessed the association between various factors and the occurrence of TS while adjusting for intrapatient correlations, are presented in Table 3. The analysis identified four factors that were significantly associated with the presence of TS (p < 0.05): age, WFNS grade, visual complaints and posterior globe thickness.

**Table 3:** Univariate analysis of factors associated with TS occurrence. Statistical significance was set at p < 0.05 for all tests. TS: Terson syndrome; WFNS: World Federation of Neurosurgical Societies

Factor	Category/Unit	Odds Ratio (OR)	95% Confidence Interval (CI)	p-value
Age	Per 1-year increase	0.86	0.78−0.96	0.005
WFNS Grade	High Grade (IV-V) vs. Low Grade (I-III)	6.02	1.30−27.78	0.021
Modified Fisher Scale	High Risk (3-4) vs. Low Risk (0-2)	2.22	0.22−22.67	0.500
Seizures	Presence vs. Absence	1.88	0.67−5.29	0.233
Aneurysm Location	Anterior Circulation vs. Posterior Circulation	1.17	0.18−7.44	0.870
Complaint of Visual/Visual Field Impairment	Presence vs. Absence	7.21	1.35−38.57	0.021
Posterior Globe Thickness	Per 1-mm increase	8	1.12−57.40	0.038

Age

An increase of one year in age was associated with a significant decrease in the odds of having TS (Odds ratio (OR) = 0.86; 95% confidence interval (CI): 0.78-0.96; p=0.005).

WFNS Grade

Patients with a high WFNS grade (IV-V) had 6.02 times higher odds of developing TS than those with a low grade (I-III) (OR = 6.02; 95% CI: 1.30-27.78; p=0.021).

Visual Complaints

The presence of subjective complaints related to visual acuity or visual field defects was associated with 7.21 times higher odds of a TS diagnosis (OR = 7.21; 95% CI: 1.35-38.57; p=0.021).

Posterior Globe Thickness

For every 1-mm increase in the posterior globe thickness measured on CT, the odds of having TS increased by a factor of 8.00 (OR = 8.00; 95% CI: 1.12-57.40; p=0.038).

In contrast, the modified Fisher grade (p=0.500), presence of seizures (p=0.233), and aneurysm location (p=0.870) did not show a statistically significant association with occurrence.

Multivariate GEE logistic regression analysis

Multivariate GEE analysis was performed to identify independent predictors of TS. We included four variables that demonstrated a p-value of less than 0.20 in the univariate analysis: age, WFNS grade, visual complaints, and posterior globe thickness. An initial assessment of multicollinearity revealed no issues, with all VIF values less than 5. Using a backward elimination method based on the Wald χ2, we derived the final multivariate model (QIC = 41.45). The final model identified age and posterior level as the only significant independent predictors of TS. The results of both the multivariate and univariate GEE analyses for the independent predictors of TS are summarized in Table 4. After adjusting for other factors, the analysis showed that for each one-year increase in age, the odds of having TS decreased by 15% (adjusted odds ratio (AOR) = 0.85; 95% CI: 0.75-0.96; p=0.007). Conversely, for each 1-mm increase in posterior globe thickness, the odds of having TS increased 13.74-fold (AOR = 13.74; 95% CI: 1.35-139.76; p =0.027).

**Table 4:** Multivariate/univariate GEE analysis for independent predictors of TS. Statistical significance was set at p < 0.05 for all tests. GEE:* *Generalized estimating equation; TS: Terson syndrome

	Parameter	Change per Unit	B	Adjusted Odds Ratio (AOR)	95% Confidence Interval (CI)	p-value
Multivariate Model	Posterior Globe Thickness	Per 1 mm increase	2.62	13.74	1.35ｰ139.76	0.027
	Age	Per 1-year increase	-0.167	0.85	0.75ｰ0.96	0.007
Univariate Model	Posterior Globe Thickness	Per 1 mm increase	2.08	8	1.12ｰ57.40	0.038

Sensitivity check 

The main findings were confirmed through a patient-level logistic regression analysis (n=26) adjusting for age. In this sensitivity analysis, the mean posterior globe thickness remained a significant independent predictor of any-eye TS (OR = 1799.02; 95% CI: 4.68-6.91×10⁵; p=0.014), while age showed a trend toward protection (OR = 0.78; 95% CI: 0.59-1.03; p=0.081). This consistency demonstrates the robustness of the primary eye-level GEE model's conclusion.

Evaluation of predictive performance by ROC curve analysis

**Figure 2:**
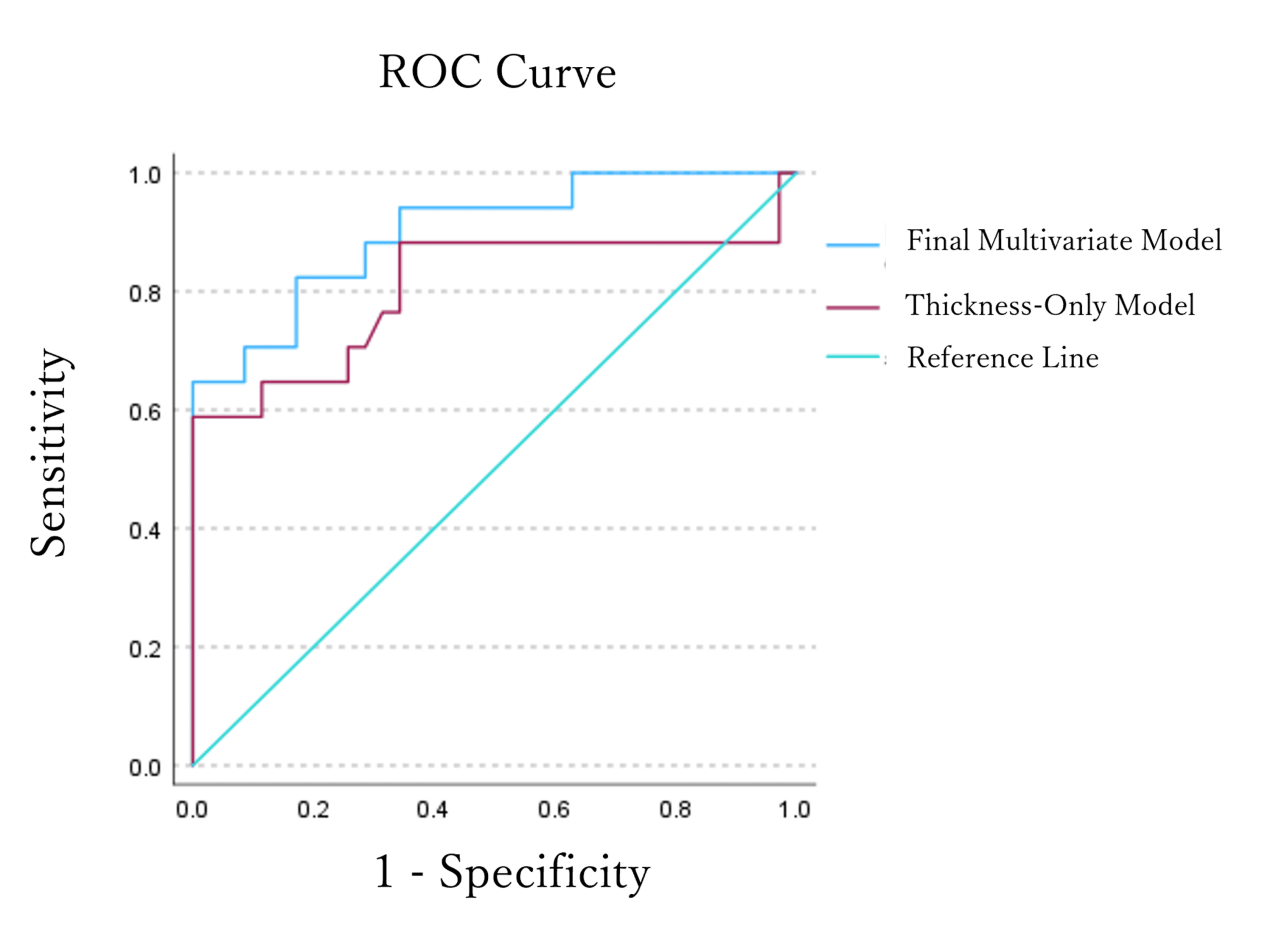
Terson syndrome (TS) predictive performance based on receiver operating characteristic (ROC) analysis. The final multivariate model (AUC = 0.901) showed superior predictive capability for TS compared to the thickness-only model (AUC = 0.806).

The predictive capabilities of the models were evaluated using the area under the receiver operating characteristic curve (AUC), as shown in Figure 2. The final multivariate model achieved an AUC of 0.901 (95% CI: 0.809-0.992), demonstrating superior predictive performance compared to the model based on posterior globe thickness alone (AUC = 0.806). The optimal cutoff values and their corresponding diagnostic thresholds for thickness are outlined below.

Thickness-Only Model

The optimal cutoff probability was 0.50080, which yielded a sensitivity of 58.8% and specificity of 100%. This probability corresponds to an estimated posterior globe thickness of 2.30 mm.

Final Multivariate Model

The optimal cutoff probability was 0.33271, providing a balanced sensitivity of 82.4% and specificity of 82.9%. The diagnostic thickness threshold derived from this model is age dependent and can be calculated using the following formula:

Cutoff Thickness (mm)≈−1.295+(0.0637×Age in years)

## Discussion

TS is a critical complication of aSAH that can significantly affect functional outcomes, and delay in its diagnosis can adversely affect the rehabilitation process [13]. Therefore, not only neurosurgeons, but also intensivists and radiologists involved in the care of patients with aSAH should be recognized. However, in clinical practice, TS is often initially overlooked and diagnosed only after a patient reports visual acuity or visual field disturbances [18-23]. A primary reason is that many patients with aSAH present with impaired consciousness, rendering them unable to articulate their symptoms accurately. Furthermore, even when aware of this condition, neurosurgeons may fail to detect it because of its rarity. Among the TS cases in our study, very few were initially suspected by the treating neurosurgeons. The gold standard for diagnosing TS is a formal ophthalmological examination [3,7], and the lack of diagnostic tools for non-ophthalmologists contributes to this challenge.

Although CT findings, such as semilunar or nodular hyperdense areas on the retina, have been described for TS [16], their diagnostic utility is limited. Bäuerle et al. reported that while ophthalmic ultrasound showed 100% sensitivity and specificity for detecting vitreous and/or preretinal hemorrhage compared with fundoscopy, head CT only had a sensitivity of 60% and a specificity of 96% [14]. Similarly, Koskela et al. found that the diagnostic sensitivity of CT for TS was only 42% with a specificity of 97% [17]. The low sensitivity of these reports highlights the tendency for missed diagnoses using CT. Moreover, the criteria for these imaging findings are often ambiguous, making their interpretation difficult. To address this gap, our study aimed to propose a threshold for posterior globe thickness to serve as a practical triage tool for neurosurgeons, enabling the accurate guidance and prioritization of patients requiring formal ophthalmologic evaluation for TS. This goal was achieved by measuring the posterior globe thickness on CT scans, an imaging modality that virtually all patients with SAH requiring treatment undergo, and by defining a cutoff value with high sensitivity and specificity.

This study had an inherent selection bias owing to its retrospective, single-center design and specific criteria for ophthalmological consultation (i.e., symptoms or physician judgment). Although the analyzed group was significantly younger than the non-analyzed group, there were no significant differences in key prognostic factors related to SAH severity, such as the WFNS and modified Fisher grades. This suggests that the selection was not driven by hemorrhage severity but rather that the sample was reasonably representative of the broader aSAH population. Our findings are consistent with those of Hong et al., who reported a higher incidence of TS in younger patients [24]. This implies that the age difference observed in our study may reflect an age-related bias, wherein the at-risk younger demographics were preferentially selected for examination. We adjusted for this confounding factor by incorporating age in the multivariate GEE model. The subsequent identification of the posterior globe thickness as a strong independent predictor of TS, even after this adjustment, supports the validity of our primary findings, despite the initial selection bias.

The predictive power of our model improved significantly from an AUC of 0.806 for the thickness-only model to 0.901 for the final multivariate model that combined age and thickness. This underscores that a composite assessment integrating a noninvasive imaging marker (thickness) with a key clinical variable (age) substantially enhances diagnostic certainty compared to relying on a single metric. A comparison of the optimal cutoff values is clinically relevant. The 2.30 mm cutoff from the thickness-only model, while achieving 100% specificity, had a sensitivity of only 58.8%, which is comparable to that in previous reports [14]. Although it is useful for ruling out TS, it carries a high risk of missing cases. In contrast, our final multivariate model, which calculates an age-dependent thickness threshold, achieved a well-balanced and high diagnostic performance with a sensitivity of 82.4% and specificity of 82.9%. This balanced approach provides a reliable clinical standard that minimizes the risk of missed diagnoses, while avoiding excessive overdiagnosis. It serves as a noninvasive screening tool applicable to all patients with SAH undergoing CT.

In settings where performing ophthalmologic examinations on all patients with SAH is challenging owing to resource constraints [11], our proposed diagnostic criteria can help triage patients, ensuring that those at a high risk of TS receive timely specialist consultations. This aligns with the approach suggested by Stewart et al., who proposed that only patients with SAH with specific CT findings (semilunar or nodular hyperdensities) should undergo ophthalmological evaluation, thereby improving cost-effectiveness [25]. The diagnostic value of our model is particularly high in younger patients whose visual and functional prognoses can be significantly improved with early intervention and rehabilitation. Furthermore, because visual impairment can lead to underestimation of a patient's level of consciousness [26,27], early TS detection may prevent such misinterpretations.

The observation that WFNS grade and visual complaints, although significant in the univariate analysis, lost their significance in the multivariate model suggests that they may be confounded by age and posterior globe thickness. Alternatively, this may indicate that the posterior globe thickness is a more direct pathophysiological marker of acute events leading to TS. The most widely accepted mechanism of TS is a sudden increase in intracranial pressure from SAH, which increases retinal venous pressure and causes intraocular hemorrhage [3]. Our measurements were obtained from an initial CT scan, supporting the hypothesis that the acute pressure surge was an inciting event. Stewart et al. suggested that most intraocular hemorrhages originate from the superficial and deep retinal capillary plexuses, often away from the optic nerve [25]. This is consistent with our observation that the point of maximum posterior globe thickness does not always coincide with the optic nerve location.

In addition to CT, MRI may also aid in the diagnosis of TS. Posttreatment MRI is common in SAH management, and several case reports have described intraocular MRI findings in patients with TS [19,28]. In the context of detecting retinal hemorrhages in pediatric cases of abusive head trauma, standard MRI showed a sensitivity of 75%, which increased to 83% with high-resolution susceptibility-weighted imaging (SWI) of the orbit [29]. In our study, SWI was not performed; however, gradient-recalled echo (GRE) sequences were acquired routinely. Among the six eyes that required surgical treatment, five showed GRE findings suggestive of hemosiderin deposition. However, a practical limitation of MRI in the postoperative SAH setting is the motion artifact, which often degrades image quality. Therefore, the shorter acquisition time of CT makes it a more robust and practical screening tool for TS than MRI.

This study has some limitations that warrant careful consideration when interpreting and generalizing the results. First, its single-center retrospective design inherently limited the external validity of the findings. Second, there are multiple sources of potential selection bias. The study population was restricted to 177 patients who received definitive treatment for their aneurysms, excluding the most severe SAH cases in which the patients died before the intervention. As TS is strongly correlated with SAH severity, this exclusion may have resulted in an underestimation of the true prevalence of TS, limiting the applicability of our findings to patients with SAH who are sufficiently stable for treatment. Furthermore, the analyzed cohort (n=26) was selected based on subjective complaints or physician judgment, resulting in a group that was significantly younger than the non-examined cohort. Although we argue that this reflects age-related susceptibility bias, it remains a limitation. We attempted to mitigate this by employing a GEE model to account for intra-patient correlations and by statistically adjusting for age in the multivariate analysis. The stability of the posterior globe thickness as an independent predictor after this adjustment supports the statistical robustness of our conclusions.　However, the wide confidence intervals observed reflect the limited statistical power due to the small number of TS cases, which necessitates caution when interpreting the magnitude of the effect. Finally, a limitation of the GEE model is that it estimates population-averaged effects and is less suited for predicting risk changes in a specific individual. Our back-calculation of the thickness cutoff value was based on the mean age of the cohort, and caution should be exercised when applying this specific threshold to patients of different ages. Future multicenter prospective studies are required to overcome these limitations. Such studies should include all patients with SAH to validate the pathophysiological significance of CT-based metrics and to confirm the generalizability of the diagnostic cutoff values proposed herein.

## Conclusions

This study proposes a noninvasive prediction model for estimating the presence of TS. This model serves as a practical triage tool by using posterior globe thickness measurements derived from routine initial head CT scans obtained during the diagnosis of aSAH, allowing for risk estimation by non-ophthalmology medical staff. The inclusion of age and posterior globe thickness significantly enhanced the diagnostic performance of the model. Applying this tool could mitigate the risk of missing TS and may be particularly valuable for patients unable to report visual impairment due to an altered level of consciousness. However, as this was a single-center, retrospective study, the results warrant further validation. To ensure broad generalizability and application in clinical practice, prospective multicenter trials are required to investigate and confirm these preliminary findings.
